# Permanent neonatal diabetes mellitus in China

**DOI:** 10.1186/1687-9856-2015-S1-P5

**Published:** 2015-04-28

**Authors:** Ke Huang, Li Liang, Jun-Fen Fu, Guan-Pin Dong

**Affiliations:** 1 Department of Endocrinology, Children’s Hospital of Zhejiang University School of Medicine, Hangzhou, Zhejiang, China

## Aims

Permanent neonatal diabetes mellitus (PNDM) is a rare disease which is defined as the onset of diabetes before the age of 6 months with persistence through life. Patients with KCNJ11 or ABCC8 gene mutations have the opportunity to switch to oral sulfonylurea therapy. There were limited studies about the genetic analysis and long term follow-up of PNDM.

## Method

Report four cases of PNDM, including their genetic mutations, treatments and long-time follow-ups. All of the patients and their parents got gene analysis include INS, KCNJ11 or ABCC8 gene.

## Results

None of the patients and their parents suffered from any genetic mutations of these three common genes. One of the children got continuous subcutaneous insulin infusion (CSII) and the others got multiple injections of insulin (MII). The PNDM patients had persisted after 35 months to 60 months of follow-up, 3 patients maintained almost stable blood sugar level, and 1 patient had poor sugar control.

## Conclusion

All of PNDM patients are suggested undergo genetic evaluation. For patients without KCNJ11 and ABCC8 gene mutation, oral sulfonylurea might not be considered. CSII is a useful tool for overcoming the difficulties of diabetes, and can also improve quality of life.

